# Proposed new grading of oral submucous fibrosis based on cheek flexibility

**DOI:** 10.4317/jced.51378

**Published:** 2014-07-01

**Authors:** Santosh Patil, Sneha Maheshwari

**Affiliations:** 1 Dept of Oral Medicine and Radiology, Chattisgarh Dental College and Research Institute, Rajnandgaon (Chattisgarh); 2 Dental Practitioner, Jodhpur (Raj), India

## Abstract

Objectives: Oral submucous fibrosis (OSMF) is a potentially malignant disorder of oral cavity, pharynx and upper digestive tract, characterized by progressive inability to open the mouth. Based on clinical and/or histopathological features, various classifications and grading systems have been put forth till date. The aim of the present study was to study the variance in cheek flexibility in OSMF patients, the observance of which led to the introduction of a new grading for the condition. Material and Methods: The study included 412 patients with OSMF attending to the Department of Oral Medicine and Radiology during the period from December 2008 to June 2013. A detailed history and examination of the patients was performed with special emphasis on measuring cheek flexibility. Statistical analysis was done using Chi-square test and p<0.05 was considered to be statistically significant. Results: The most common etiological factor was tobacco (73.3%). All the patients reported with burning sensation of the mouth and blanching of the mucosa. Malignancy was reported in only 4.6% patients. 60.4% patients showed cheek flexibility of 30mm and above, while 29.6% patients showed cheek flexibility between 20-30mm and 10% patients showed less than 30mm of cheek flexibility. Conclusion: The observations of the present study have led to the proposal of a new grading of OSMF based on cheek flexibility which could assist in the categorization of the condition by the clinicians.

** Key words:**Cheek flexibility, oral submucous fibrosis, grading, areca nut.

## Introduction

Oral submucous fibrosis [OSMF] is a chronic debilitating disease of the oral cavity characterized by inflammation and progressive fibrosis of the submucosal tissues (1). Pindborg and his associates defined the condition as “an insidious chronic disease affecting any part of the oral cavity and sometimes pharynx. Although occasionally preceded by and/or associated with vesicle formation, it is always associated with juxtaepithelial inflammatory reaction followed by fibroelastic changes in the lamina propria, with epithelial atrophy leading to stiffness of the oral mucosa causing trismus and difficulty in eating (2). Susrutha in ancient medicine described a condition similar to OSMF as “vidari”, under the umbrella of mouth and throat diseases (3). In 1952, Schwartz (4) described a condition of the oral mucosa as “atrophia idiopathica mucosa oris”, with the term OSMF coined by Joshi in 1953 (5). The pathogenesis of the disease is not well known, but the etiology is believed to be multifactorial. The condition is particularly associated with areca nut chewing, which is the main component of betel quid. The habit of betel quid chewing is practiced predominately in the Indian subcontinent from a long time (1).

Several classifications, staging and grading systems based on clinical and histopathological features and other aspects of OSMF have been put forth by various researchers. The advantages and disadvantages of these systems supersede the other leading to confusion. Till date no grading system for OSMF has been proposed on the basis of flexibility of cheek, so a new system has been proposed to for grading the OSMF according to the cheek flexibility.

## Material and Methods

This study included 412 patients with OSMF attending the Department of Oral Medicine and Radiology, Jodhpur Dental College General Hospital, Jodhpur National University, Jodhpur, India. Patients who were consuming areca nut and its products and who were clinically exhibiting signs and symptoms of OSMF were included in this study. Patients with any systemic diseases were excluded from the study. The study was conducted from December 2008 to June 2013. Ethical clearance was obtained from the institutional ethical committee. A written informed consent was obtained from the patients prior to the study. A detailed personal history of the patient regarding use of the areca nut and its products, consumption of chilly, spicy food, consumption of tobacco and alcohol was recorded. Complaints regarding burning sensation, limited mouth opening, and difficulty in speech, swallowing, hearing, dryness, excess salivation, ulcers, vesicles and taste change with their duration were noted. Patients were examined for blanching of oral mucosa, presence of fibrous bands, ulcers, vesicles, erosions, status of the uvula and concurrent presence of other premalignant and malignant conditions. Mouth opening and tongue protrusion were measured as previously described. Cheek flexibility was measured as the distance, in millimeters, from the maxillary incisal midline to the cheek retractor during retraction (6). Normal cheek flexibility as observed in males was 35-45 mm and 30-40 mm in females. Presence of any precancerous condition, lesion or any malignancy was confirmed by biopsy. The observations were analyzed using the computer program, SPSS 12 [SPSS Inc. Chicago, USA]. Statistical analysis was done using Chi-square test and p<0.05 was considered to be statistically significant.

## Results

In the present study, out of 412 OSMF patients, there were 220 males and 192 females, with an age range of 18-56 years. The mean age of the patients was 27.8±8.6 years. The various predisposing factors in the patients were identified and areca nut [73.3%] was the most common etiological factor followed by tobacco [69.2%] ( Table 1). Tobacco was the most common etiological factor in males while areca nut was the most common etiological factor in females. The various symptoms as reported by the patients are shown in  Table 2. All the patients complained of burning sensation. 85.1% patients reported difficulty in mouth opening, whereas 56.8% patients reported change in taste. Dryness of the mouth was reported in 27.4% patients while 53.6 patients complained of excessive salivation. Vesicles and ulcers were reported in 14.8% patients. The signs as observed by the examiners are shown in  Table 3. The most common sign observed was blanching of the mucosa, which was reported in all the patients. Fibrous bands were reported in 94.6% patients. 285 patients showed trismus and 100 patients showed restricted tongue movements. Deviated uvula was observed in 62 patients. 31.3% patients showed signs of leukoplakia and 15.3% patients had lichen planus. Only 19 patients showed signs of malignancy. Males were commonly more affected than females and this difference was statistically not significant [p>0.05]. The distribution of the patients according to cheek flexibility has been shown in  Table 4. 60.4% patients showed cheek flexibility of 30mm and above, while 29.6% patients showed cheek flexibility between 20-30mm and 10% patients showed less than 20mm of cheek flexibility.

**Table 1:**
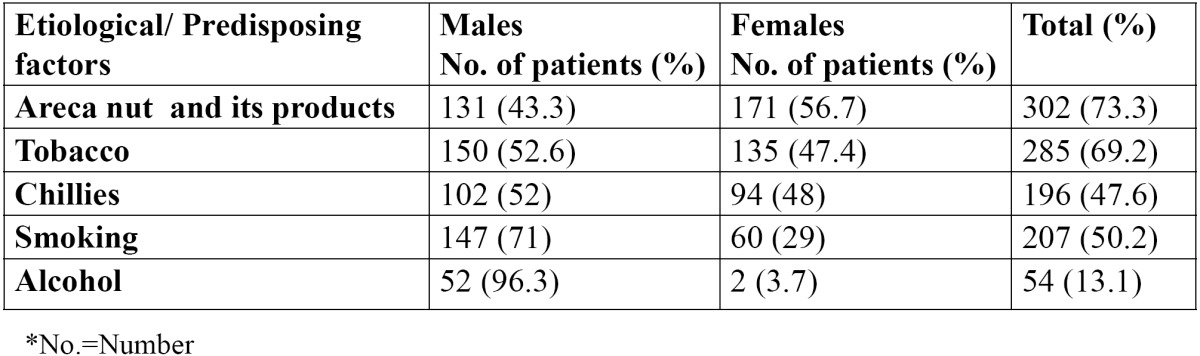
Distribution of various etiological factors

**Table 2:**
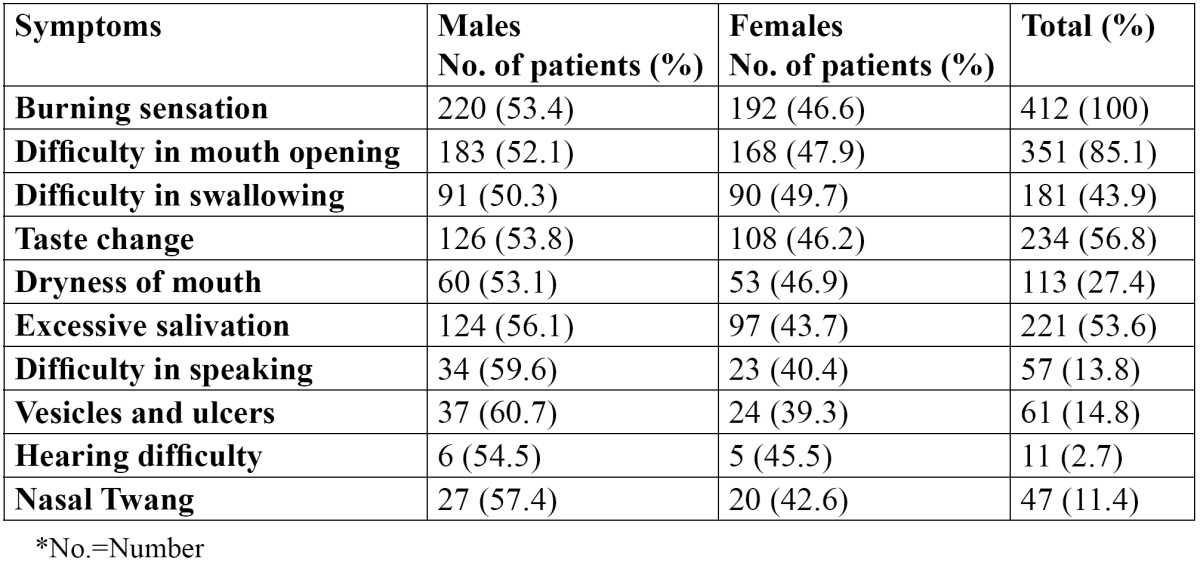
Distribution of the various symptoms in OSMF patients.

**Table 3:**
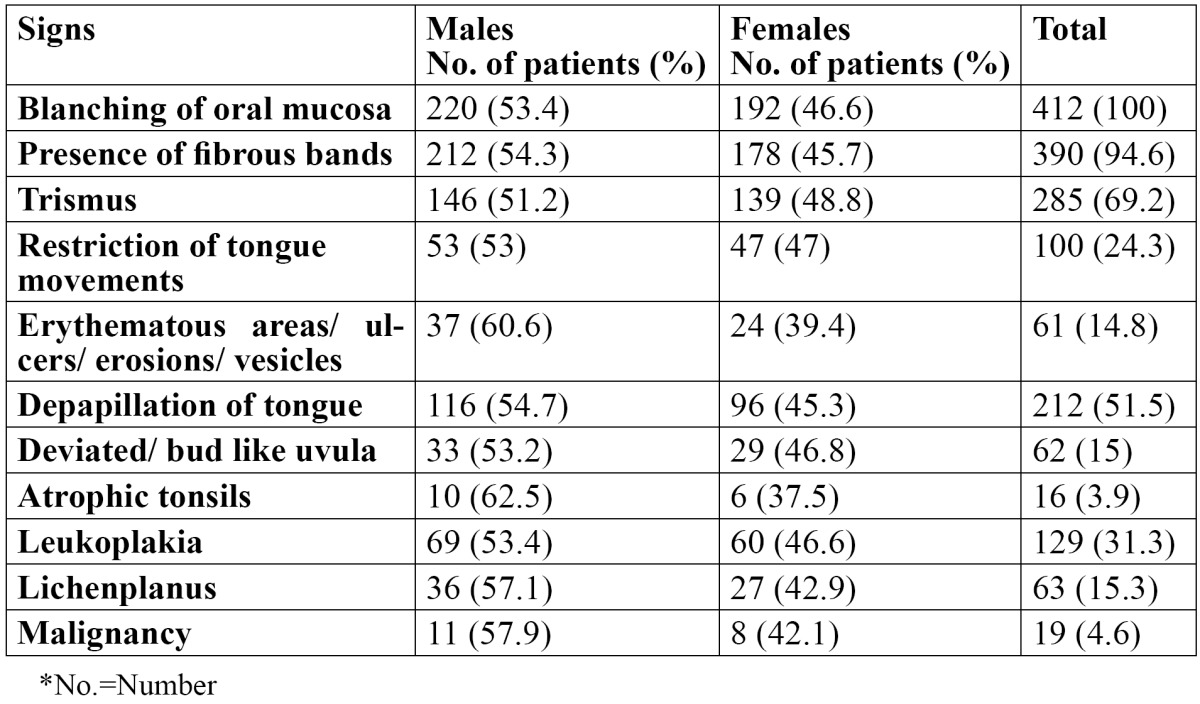
Distribution of the various signs in OSMF patients.

**Table 4:**
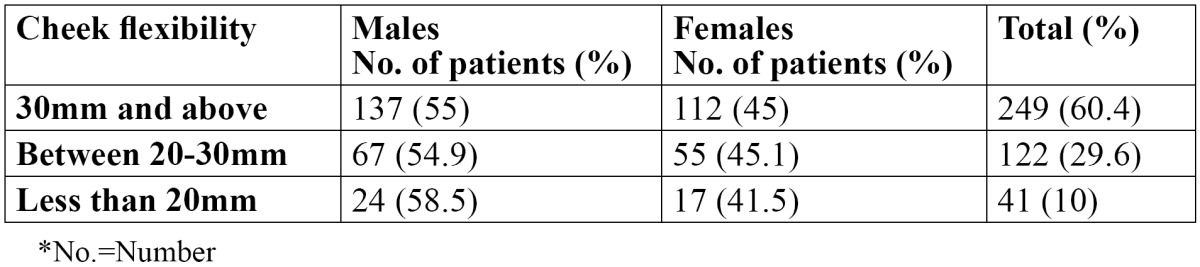
Distribution of patients according to cheek flexibility.

## Discussion

OSMF is a precancerous condition of the oral cavity and oropharynx which is predominantly seen in the Indian subcontinent and Southeast Asian countries and is now globally considered an Indian disease. The overall prevalence rate in India is believed to be about 0.2% to 0.5% and prevalence by gender varying from 0.2-2.3% in males and 1.2-4.57% in females (7,8). It is considered to have a high degree of malignant potential, which ranges between 2.3% and 7.6% (9). The pathophysiology of OSMF is complex, and various factors such as, ingestion of chilies, genetic susceptibility, nutritional deficiencies, altered salivary constituents, autoimmunity and collagen disorders may be involved in the pathogenesis (7). Areca nut and its products [73.3%] was the most common etiological factor in the present study. The condition is preceded by burning sensation of the oral mucosa, ulceration and pain. OSMF is characterized by blanching of oral mucosa, reduced movement and depapillation of tongue, depigmentation of oral mucosa, and progressive reduction of mouth opening (10,11). Nasal twang due to fibrosis of nasopharynx and hearing impairment due to stenosis of eustachian tube may be observed in advanced stages of the condition. Majority of OSMF patients present with irreversible moderate-to-severe condition. The changes of OSMF are limited to oral tissues and similar to those of scleroderma. It may be associated with oral leukoplakia and other potentially malignant disorders or with malignancy such as squamous cell carcinoma (10). The higher prevalence of leukoplakia [31.3%] in OSMF leading to oral carcinoma was observed similar to the finding of Thukanaykanpalayam *et al.* (8). The precancerous nature of OSMF has been proved by, higher occurrence of OSMF in oral squamous cell carcinoma patients, histological diagnosis of cancer without any clinical suspicion in OSMF, high frequency of epithelial dysplasia and higher prevalence of leukoplakia among OSMF. The debate over the initiation of malignancy in OSMF due to epithelium or due to connective tissue is still unanswered (8,12). However, it has been suggested that the pathology develops within the epithelium due to intraoral trauma and various factors such as, irritation from jagged teeth, sharp overhanging restoration, ill-fitting dentures, jacket crowns, prolong use of tobacco and poor oral hygiene (8,13). Only 19 patients showed signs of malignancy in the present study.

Treatment modalities for relieving the symptoms have been advocated, but have not been successful so far. The first step of preventive measure should be in discontinuation of habit, which can be encouraged through education, counseling and advocacy. Medical treatment is symptomatic and predominantly aimed at improving mouth movements. But each treatment has its own limitations (14).

Based on the features of OSMF, such as clinical symptoms, maximal mouth opening, and palpable fibrous bands, many researchers have divided it into different clinical stages. Pindborg JJ (15) reviewed the first clinical classification of OSMF based on the physical findings of the disease. But this classification did not include the mouth opening of the patients. Ahuja and Agrawal (16) classified submucous fibrosis clinically based on the extent and type of fibrosis.

Lai DR *et al.* (17) in 1995 classified OSMF on the basis of mouth opening but clinical symptoms of patients were not considered in this classification. Khanna and Andrade (18) categorized OSF into different stages considering the clinical features, histological features and mouth opening of the patients. Chandramani More *et al.* (10) in 2011 provided a clinical staging of OSMF considering the symptoms of the disease and presence of the palpable fibrous bands. None of the above mentioned researchers considered check flexibility in grading or staging of OSMF. Hence, through the results of the present study, OSMF can be graded as follows based on cheek flexibility:

. Grade 1 [Early]: Cheek flexibility of 30 mm and above

. Grade 2 [Mild]: Cheek flexibility between 20-30 mm

. Grade 3 [Moderate]: Cheek flexibility less than 20 mm

. Grade 4 [Severe]: Any of the above condition without concurrent presence of potential malignant lesions

. Grade 5 [Advanced]: Any of the above condition with concurrent presence of oral carcinoma

An attempt has been made to propose a new grading system for OSMF, to assist the practitioners and researchers in the categorization of this premalignant condition and to aid in early detection thereby leading to timely management.
